# Lumbar hemivertebra resection in congenital scoliosis utilizing cone-beam navigation: less radiation, more accuracy—proof of concept

**DOI:** 10.1007/s00381-021-05055-5

**Published:** 2021-01-28

**Authors:** Christian Fisahn, Chris Lindemann, Brittni Burgess, Patrick Strube, Timo Zippelius

**Affiliations:** 1 Department of Orthopedics, Jena University Hospital, Waldkliniken Eisenberg, Campus Eisenberg, Eisenberg, Germany; 2 University of Colorado School of Medicine, Anschutz Medical Campus, Denver, CO USA

**Keywords:** Hemivertebrae, Congenital scoliosis, Vertebral column, Spine, Intraoperative CT, Navigation, Cone-beam navigated system (CBNS)

## Abstract

**Purpose:**

To present the first known pediatric utilization of cone-beam navigation system (CBNS) for hemivertebra resection and spondylodesis

**Case presentation:**

A 14-year-old female with congenital scoliosis, diagnosed at 8 years of age, presenting with progressive symptoms, a Cobb angle (L3-5) of 38° at time of surgery, treated historically with conservative measures. Presence of spinal intramedullary disease was excluded prior to operation via whole spine MRI.

**Results:**

Patient successfully underwent surgical correction utilizing the CBNS (O-arm™, Medtronic®). Post-operative Cobb angle (L3-5) was restored to 8°. Following four different pediatric patient’s radiation exposures (two receiving correction via the O-arm platform and two via the traditional method employing fluoroscopy), we show a reduction in radiation exposure using the CBNS system.

**Conclusion:**

We present the first known pediatric case of the utilization of the CBNS system for hemivertebra correction. We demonstrate that utilizing the CBNS platform can not only increase surgical accuracy but also decrease pediatric patient’s radiation exposure as a preoperative CT scan is not needed. Future studies should continue to explore additional benefits of implementing the system into surgical practice.

## Introduction

Hemivertebra is a vertebral anomaly due to the lack of formation of a vertebral body that can result in abnormal curvature of the spine. It can give rise to multiple congenital spinal deformities and is most commonly associated with congenital scoliosis [1, 2]. Hemivertebrae typically occur in the lumbar and thoracic spine and can progress into spinal disorders, such as congenital kyphosis, pain, and loss of function, if not surgically treated during infancy.

Prompt diagnosis and treatment of hemivertebra are essential to avoid progression of spinal deformity and to reduce the potential damage to adjacent segments [3]. A variety of surgical procedures exist for hemivertebra correction, including but not limited to in situ fusion, convex growth arrest, anterior and posterior fusion with or without instrumentation, hemivertebra excision, and fusion via a combined anterior and posterior approach in 1 or 2 stages. Historical treatment methods require use of traditional fluoroscopy and preoperative CT scan to guide the procedure, exposing children to high radiation doses.

We hypothesized that using 3D real-time-guided navigation (O-Arm, Medtronic®) would result in a high level of accuracy as well as a significantly reduced radiation exposure to the child compared to traditional fluoroscopy.

To the best of our knowledge, this is the first pediatric presentation of the use of the CBNS for hemivertebra correction.

## Case description

**Fig. 1:**
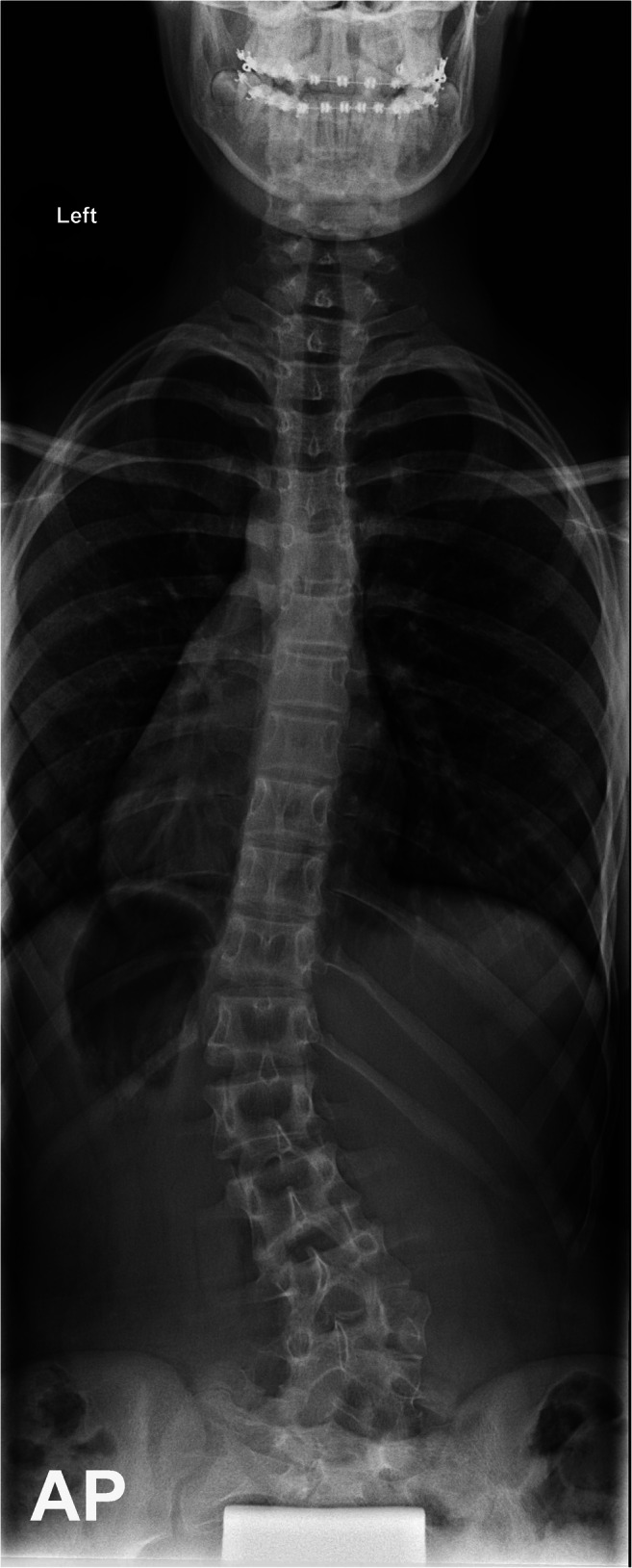
Preoperative imaging. Total spine x-ray in ap view (standing)

**Fig. 2:**
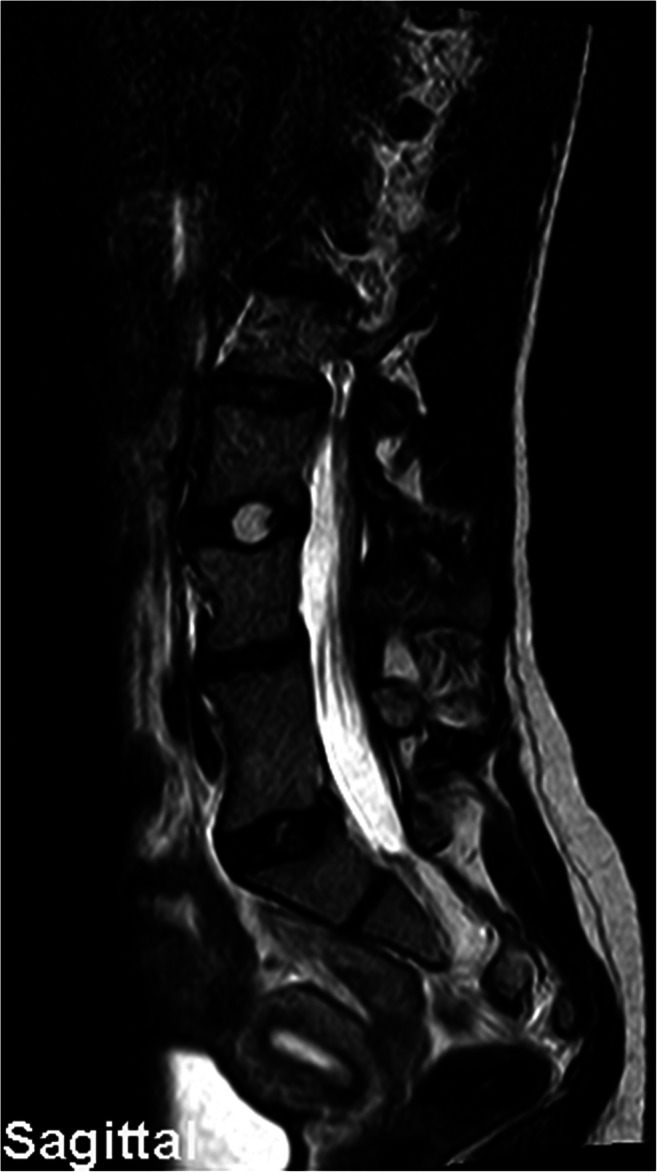
Preoperative imaging. MRI sagittal plane

An otherwise healthy female patient first presented to our outpatient clinic at 8 years of age. Her parents brought her as they noticed a visible back deformity, especially while leaning forwards. The patient reported no obvious discomfort or pain. Imaging demonstrated that the left shoulder was 1.5 cm higher than the right shoulder, and there was a single fully segmented hemivertebra with a wedge shape on the L4 junction right-sided. Segmental scoliosis (L3-L5) was 30°. The patient had a Risser grade of 0-1. Congenital scoliosis (CS) was diagnosed. She had received conservative treatment in the form of physiotherapeutic scoliosis-specific exercises and rigid bracing using a Cheneau brace. However, scoliosis had progressed over the years to a Cobb angle of 38° (Th11-L3) at 14 years of age and a BMI of 18.8 (Fig. 1). After preoperative discussion and evaluation, the girl underwent posterior 3D real-time-navigated hemivertebra resection. In magnetic resonance imaging of the thoracic and lumbar spine, the presence of spinal intramedullary disease could be excluded (Fig. 2).

## Surgical procedure

**Fig. 3:**
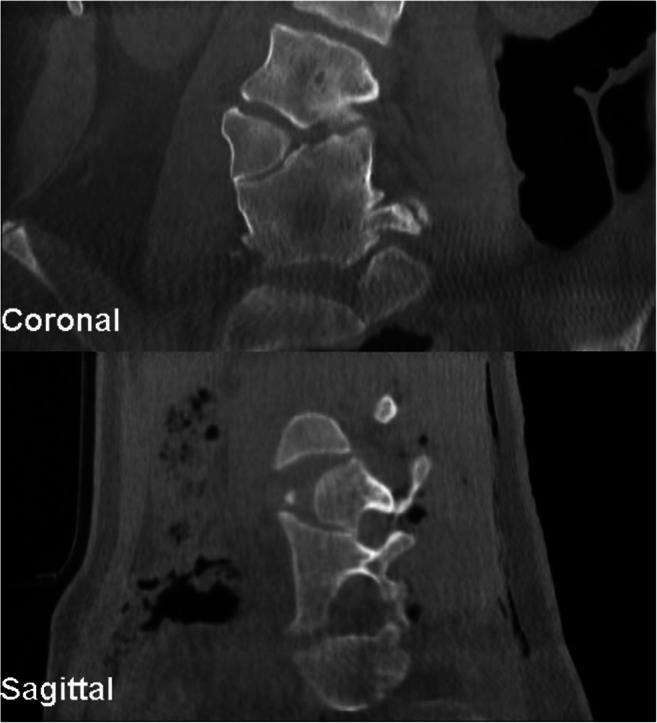
Intraoperative 3-D O-arm scan in coronal and sagittal plane

The patient was positioned prone on a radiolucent table. For 3D real-time-guided navigation, we utilized a CBNS (O-arm™, Medtronic Inc., MN, USA). The O-arm was positioned around the patient. Under the guidance of O-arm fluoroscopy, the areas of interest were marked. After aseptic skin preparation and sterile covering of the O-arm, a posterior midline skin incision was made along the marked points. And the retractors were then positioned. The navigation reference frame was affixed to the exposed caudal spinous process tip or pelvic crest. The spinal segment of interest was scanned using the O-Arm, and the images were automatically sent to the StealthStation navigation system (Medtronic Inc., MN, USA) (Fig. 3). During this process, pediatric protocols and collimation can be used to dramatically reduce patient radiation exposure [4].

**Fig. 4:**
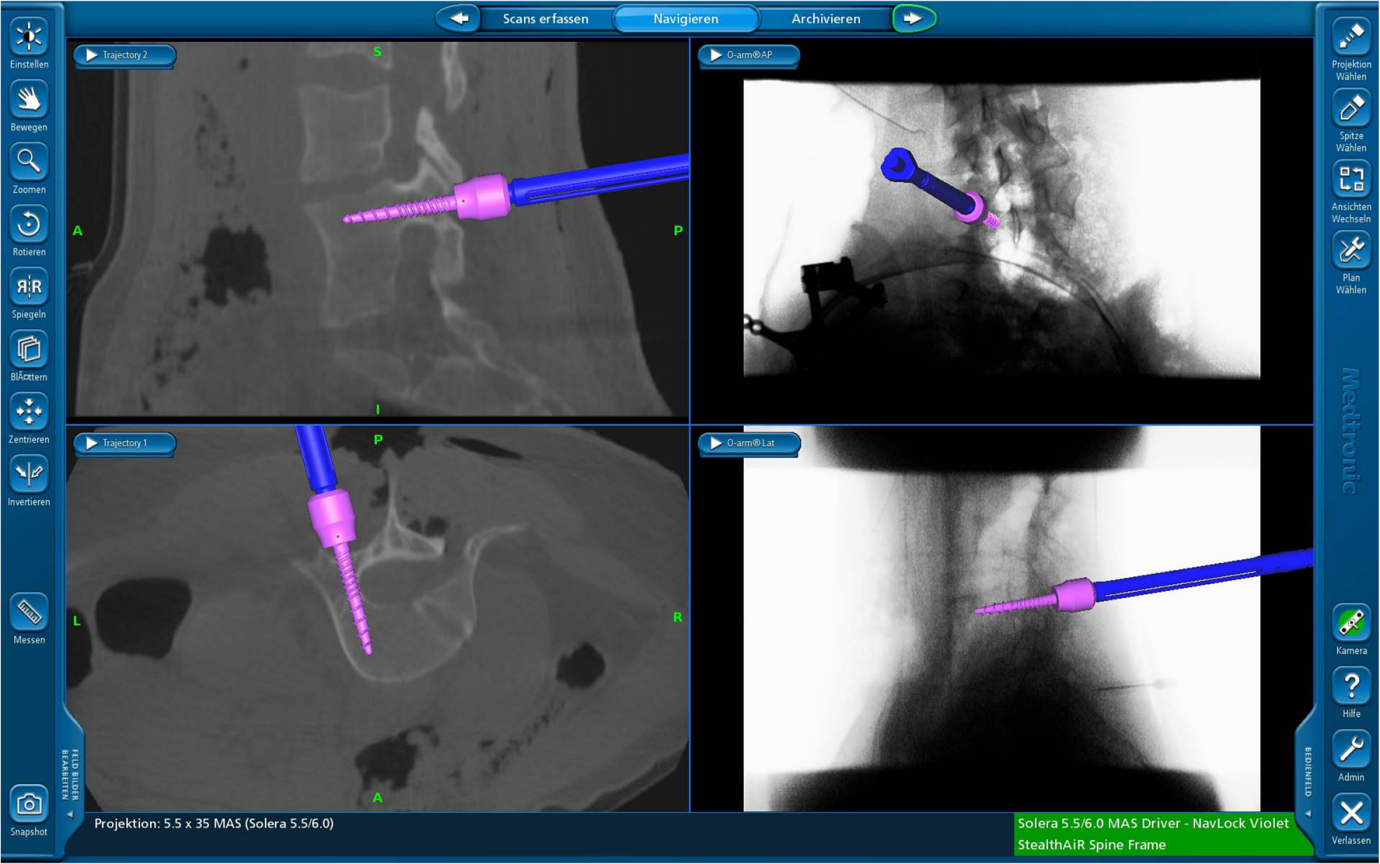
Pedicle screw placement utilizing O-arm navigation

**Fig. 5:**
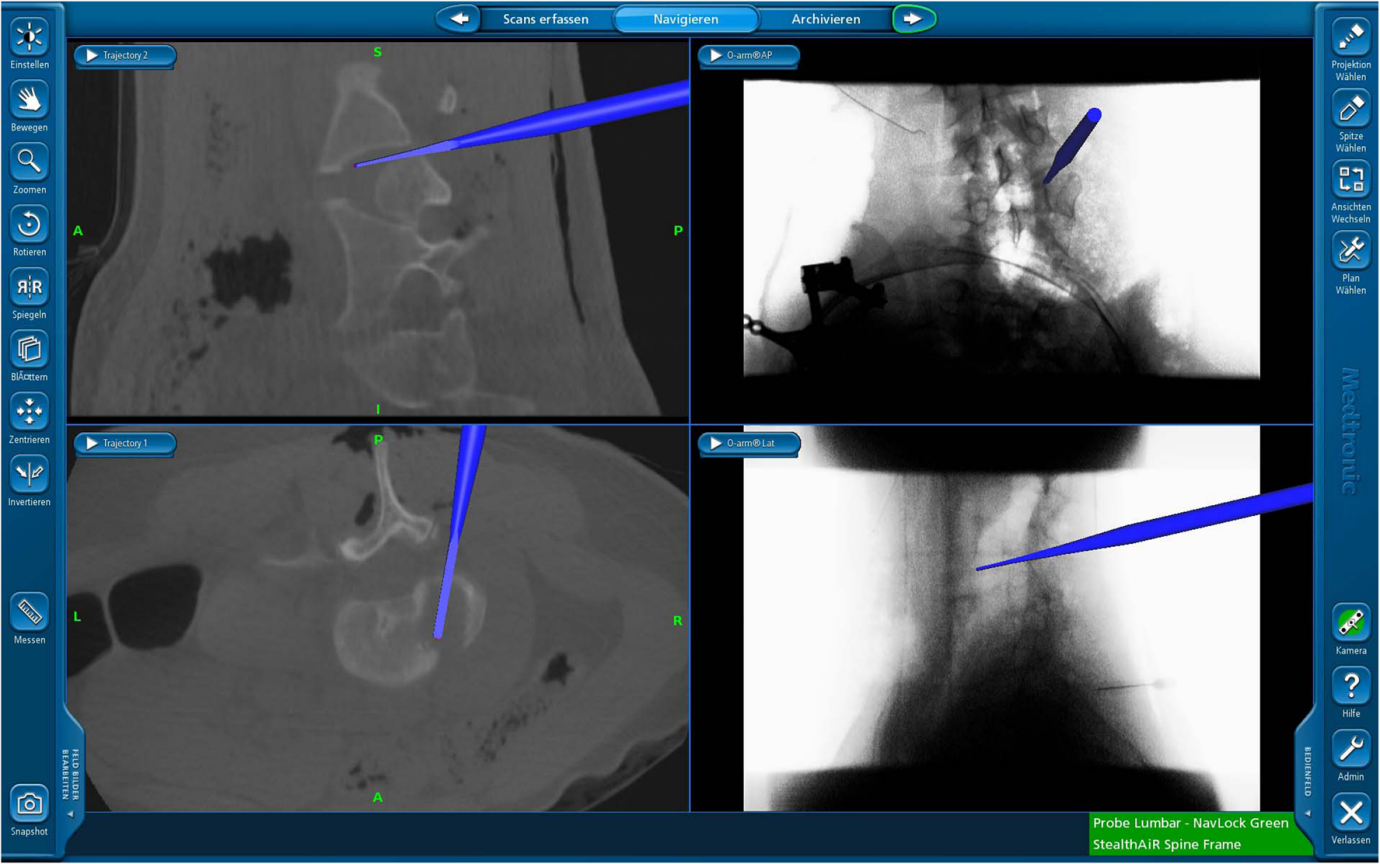
Navigated visualization of the hemivertebrae

First, the pedicles were cannulated with a navigated awl. Next, the pedicle screws (L3 and L5) were inserted with a navigated driller to the optimal entry point and trajectory (Fig. 4). Now, the posterior aspects of the vertebra of L4 such as lamina, facet joint, and pedicle are resected. The dural sac and the nerve roots above and below the pedicle of the hemivertebrae are visualized (Fig. 5). Afterwards, we inserted a longitudinal rod on the concave side for securing segment stability.

Under image guidance/navigation, the vertebral body was gradually removed with a Kerrison punch, bone curette, and bone chisels. With a navigated chisel (Medtronic Inc., MN, USA), the depth and margin of the bony structure can also be observed simultaneously in real-time by the navigation system to ensure adequate removal of the bony structure without damaging adjacent vital organs or major vessels.

**Fig. 6:**
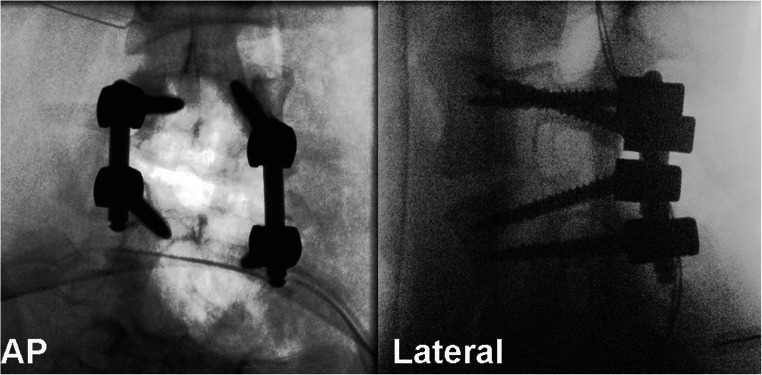
Final fluoroscopy image in ap and lateral view

Finally, rods were fixed to the pedicle screws after the reposition of the spinal segment. A sufficient amount of bone graft was placed into the posterior vertebra. The positions of screws and rods were verified through fluoroscopy, and the wounds were subsequently closed (Fig. 6) The operative time was 165 min.

**Fig. 7:**
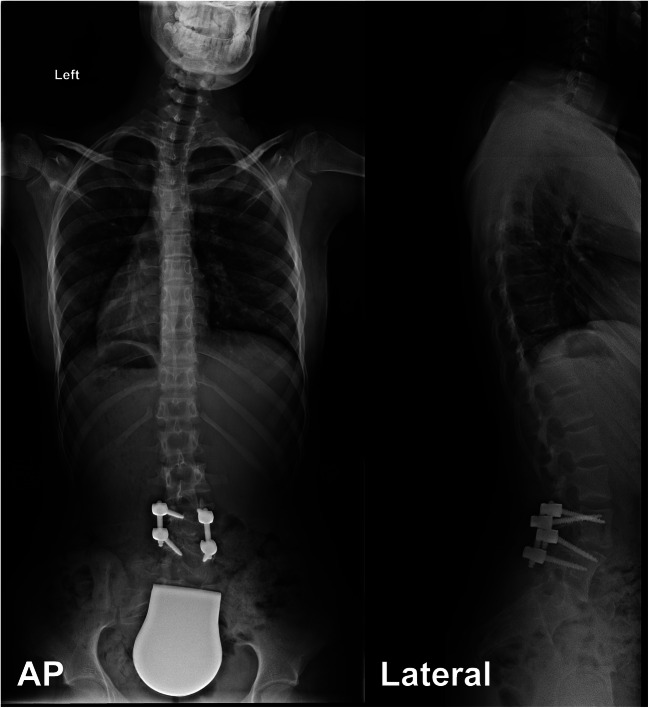
Postoperative imaging. Total spine x-ray in ap and lateral view (standing)

At postoperative follow-up, the Cobb angle (L3-5) was corrected to 8° (Fig. 7).

## Radiation exposure

In order to determine if utilizing the CBNS reduced pediatric radiation exposure, we tracked four children’s imaging experiences with hemivertebra correction to calculate radiation exposure. Two children received pre-operative CT work and intraoperative fluoroscopy, following a traditional approach. In contrast, the other two children had intraoperative 3D O-arm scans and intraoperative fluoroscopy. The effective dose for each type can be found in (Table 1). We concluded that the CBNS system is not only an accurate surgical approach, but can significantly reduce the radiation exposure children receive, as compared to traditional fluoroscopy.

**Table 1:** Radiation exposure in CBNS compared to CT and fluoroscopy

Procedure	DLP (mGycm)	ED (mSv)	DAP (cGycm^2^)	ED (mSv)	ED gesamt (mSv)	Factor CT vs. 2D
CBNS	3D Scan		Intraop, Fluoro			
Patient 1	65.89	1.074007	95.73	0.201033	**1.27504**	5.3
Patient 2	36.51	0.595	6.399	0.0134	**0.6084**	44.4
CT and fluoroscopy	CT thoracic spine		Intraop, Fluoro			
Patient 3	88.9	1.94691	90.54	0.190134	**2.137044**	10.2
Patient 4	110.8	2.42652	90.98	0.191058	**2.6175**	12.7

*DLP* dose length product, *ED* effective dose, *DAP* dose area product

## Discussion

To our knowledge, this manuscript presents the first report of using the CBNS system for lumbar hemivertebra resection. In addition to the excellent possibility of precisely inserting implants and controlling and correcting incorrect positions intraoperatively, the use of the O-arm resulted in a significant reduction in the radiation dose. It is imperative to have accurate surgical techniques for hemivertebra correction to ensure we minimize curve progression and trunk imbalance. In addition, a preoperative CT can be completely dispensed, which offers a great advantage over conventional methods such as neuronavigation.

Widespread efforts have been implemented to reduce pediatric radiation exposure due to pediatric patients being more radiosensitive, and having a longer expected lifetime, they are at a higher risk for cancer. In the last 30 years, the average radiation doses exposed to children have doubled in the USA [5]. CT is recognized as a large contributor to this rise in radiation exposure, and recent studies have found that CT scans performed during childhood (head and abdomen) result in a 10-fold increase in estimated risk compared to the same scans performed in adults. The increase in lifetime cancer mortality risk for a 1-year-old child associated with a CT scan is 0.18% and 0.07% for the abdomen and head, respectively. Throughout this paper, we demonstrated that utilizing the O-arm system can dramatically reduce radiation exposure for pediatric patients receiving hemivertebra revision. Previous work demonstrates the importance of judicious use of CT scans for pediatric patients, and implementing an O-arm system can reduce the need for pre-operative CT scans thereby reducing overall radiation exposure. The intraoperative scan using the O-arm enables a CT-like image generated in the 3D reconstruction. In this way, a precise assessment of all bony aspects can be made (Figs. 3 and 5). It is also possible to use the pointer or chisel to determine exactly the point at which resection has to be made. The surgical intervention showed excellent results with the correction over one segment. This can also be expanded if necessary.

Furthermore, this case describes an innovative approach for the treatment of congenital scoliosis due to semi-segmented hemivertebrae. This unique case study has shown the accuracy of using the O-arm system and the decreased radiation benefit of implementing such a system.

## Conclusion

Throughout this manuscript, we presented the first pediatric case utilizing the O-arm system for hemivertebrae correction and spondylodesis. We were able to show that a preoperative CT scan is not necessary, and utilization of the CBNS platform can increase accuracy of surgical procedure while decreasing radiation exposure to pediatric patients. Future studies should investigate the additional benefits of incorporating the CBNS into surgical practice for hemivertebra correction.
